# Defining Uremic Arterial Functional Abnormalities in Patients Recently Started on Haemodialysis: Combined *In Vivo* and *Ex Vivo* Assessment

**DOI:** 10.1371/journal.pone.0113462

**Published:** 2014-12-29

**Authors:** Adil M. Abushufa, Mohamed T. Eldehni, Aghogho Odudu, Philip D. Evans, Saoirse E. O′Sullivan, Chris W. McIntyre

**Affiliations:** 1 Division of Medical Sciences and Graduate Entry Medicine, School of Medicine, University of Nottingham, Nottingham, United Kingdom; 2 Department of Renal Medicine, Royal Derby Hospital, Derby, United Kingdom; University of Southampton, United Kingdom

## Abstract

Endothelial dysfunction is a key initiating event in vascular disease in chronic kidney disease (CKD) patients and haemodialysis (HD) patients exhibit significant vascular abnormalities. To understand this further, we examined how *ex vivo* intrinsic function in isolated arteries correlates with *in vivo* assessments of cardiovascular status in HD patients. Abdominal fat biopsies were obtained from 11 HD patients and 26 non-uremic controls. Subcutaneous arteries were dissected and mounted on a wire myograph, and cumulative concentration-response curves to noradrenalin, endothelin-1, a thromboxane A_2_ agonist (U46619), angiotensin II, vasopressin, bradykinin (BK), acetylcholine (ACh) and sodium nitroprusside (SNP) were constructed. Pulse wave velocity and blood pressure were measured in HD patients. Enhanced (P<0.05−0.0001) maximal contractile responses (R_max_) to all spasmogens (particularly vasopressin) were observed in arteries from HD patients compared to controls, and this effect was more pronounced in arteries with an internal diameter>600 µm. The potency (pEC_50_) of U46619 (P<0.01) and vasopressin (P<0.001) was also increased in arteries>600 µm of HD patients. The maximal relaxant response to the endothelium-dependent dilators ACh and BK were lower in HD patients (P<0.01-P<0.0001) (worse for ACh than BK); however the endothelium-independent dilator SNP was similar in both groups. PWV was significantly correlated with the vasoconstrictor response to vasopressin (*P* = 0.042) in HD patients. HD patients are primed for hypertension and end organ demand ischaemia by a highly sensitised pressor response. The failure of arterial relaxation is mediated by endothelial dysfunction. Intrinsic vascular abnormalities may be important in sensitising HD patients to recurrent cumulative ischaemic end organ injury.

## Introduction

Cardiovascular disease is the major cause of mortality in CKD patients. These patients are characterised by a wide range of both structural and functional abnormalities of the vessels. There is strong emerging evidence that the hemodialysis (HD) procedure causes significant systemic circulatory stress [1]. This circulatory stress interacts with complex haemodynamic factors causing perfusion anomalies that accelerate end organ damage in a wide range of vulnerable vascular beds [2]. Initially this work focused on the heart, but emerging evidence strongly suggests that a much wider range of organs are affected. This includes the gut, kidney, skin and critically, the brain [3]. Fixed structural abnormalities of the vascular tree develop during the lifetime of a progressive CKD patient. These include coronary calcification and other drivers of reduced arterial compliance. These changes are the result of fixed vascular structural change but importantly additional functional microvascular abnormalities are potentially superimposed onto these processes [4]. These may be more important in determining the propensity to multi organ based demand ischaemia.

It is unclear to what degree the observed abnormalities of microvascular function are related to the uremic milieu and exposure of relatively normal vasculature to a variety of functionally modifying factors, and to what degree there are intrinsic defects in pressor response. Vascular endothelium plays an important role in the regulation of vascular function and tone through production of a wide range of vasoactive substances including endothelin, nitric oxide (NO), and thromboxane A2 [5]. Different mechanisms have been proposed to cause endothelial dysfunction in HD patients, which is largely due to a defect in the endothelial NO bioavailability [6]. Several uraemia-associated factors including oxidative stress and reactive oxygen species [7], [8], homocysteine [9] and plasma nitric oxide synthase inhibitors (asymmetric dimethylarginine) [10] are found in high levels in the plasma of HD patients and may contribute to endothelial dysfunction. Impaired endothelium-dependent vasodilatation has been observed in several *in vivo* studies involving CKD [11], [12] and HD patients [13], [14]. A limited number of *ex vivo* studies [15]–[17] have examined small isolated arteries removed from uremic patients selected to be undergoing a surgical procedure have revealed similar vascular dysfunction *ex vivo* to a limited number of vasoactive agents.

Large vessel compliance (arterial stiffness), as measured by increased PWV, is higher in patients on dialysis (and with non-dialysis dependant CKD) compared with the general population [18]. Increased PWV is associated with elevated cardiovascular morbidity and mortality risks in patients with CKD stage 5 [19]. A strong association has been observed between PWV and abdominal aorta calcification in HD patients [20]. This change in compliance is partially due to material alterations in the conduit arteries, however it is also blood pressure (BP) and endothelial dysfunction related.

This current study was focused on an investigation of the vascular function in isolated resistance arteries obtained from subcutaneous fat of HD patients, with comparison to appropriate matched non uremic control. We aimed to establish whether HD patients show enhanced responses to a wider range of different vasoconstrictors and impaired endothelial-dependent vasodilatation, as previously suggested [15], [16] and how that *ex vivo* intrinsic function correlates to *in vivo* assessments of cardiovascular status. Results from smaller (internal diameter less than 600 µM) were also compared with larger arteries (internal diameter greater than 600 µM) to establish whether different calibre arteries are affected differentially by HD.

## Materials and Methods

Subcutaneous fat samples were obtained from 11 HD patients who were within the first 90 days of starting dialysis. Control fat samples were obtained from 26 appropriately consented healthy volunteers without documented renal disease, who were undergoing elective hernia repair. The ethical approval was granted by the Derbyshire Research Ethics Committee. Informed written consent was obtained in accordance with Good Clinical Practice guidelines.

Subcutaneous biopsies were obtained from HD patients using local anaesthetic (1% lignocaine) through a small (1–2 cm) lower lateral abdominal incision. Subcutaneous adipose tissue was dissected and separated from the adherent skin, and then a piece of fat was removed using scalpel and surgical scissor. Samples were placed immediately in chilled physiological salt solution (PSS) and experimented on the same day of biopsy.

### *In vitro* assessments of vascular function

Arteries of varying diameters were dissected from each sample per patient and mounted as a ring preparation on two wire myographs (ie. Up to 8 segments of different sized arteries per patient)(model M620, Aarhus, Denmark) in PSS and continuously bubbled with 5% CO2 and 95% O2. PSS was composed of (in mmol/L) NaCl 119, KCL 4.7, CaCl_2_.2H_2_O 2.5, NaHCO3 25, MgSO4.7H2O 1.17, KH_2_PO_4_ 1.18, D-glucose 5.5 and EDTA 0.026. Following an acclimatisation period, arteries were subjected to a standard normalization process [21] to determine the internal circumference of arteries. The integrity of arteries was tested by their ability to contract by at least 5 mN (0.5 g) tension to a high potassium PSS (KPSS, high potassium chloride concentration (124 mmol/L) substituted for sodium chloride). Arteries unable to contract to KPSS were discarded. Viable arteries were then contracted using 50 nM of the thromboxane mimetic U46619 and once a plateau contraction has reached, 10 µM of bradykinin was added to confirm an intact endothelium. All arteries were washed-out with PSS until re-establishment of the base line. Cumulative-concentration response curves for noradrenaline (NA, 100 pM to 100 µM), endothelin-1 (ET-1, 1 pM to 1 µM), U46619 (1 pM to 3 µM), angiotensin II (Ang II, 1 pM to 1 µM), and vasopressin (1 pM to 1 µM) were constructed in separate segments of artery. These started with the lowest concentration of the drug and allowed 3–5 minutes per stimulation.

To assess vasodilator function, separate segments of arteries were first constricted using a combination of 100 nM U46619 and 1 nM ET-1. Once the peak steady contraction had obtained, cumulative-concentration response curves for bradykinin (BK, 100 pM to 100 µM), acetylcholine (ACh, 100 pM to 100 µM) and sodium nitroprusside (SNP, 100 pM to 100 µM) were constructed.

### *In vivo* haemodynamic measurement

Brachial artery BP was measured in the non-fistula arm using an oscillometric device. Carotid-to-femoral PWV was measured using a Vicorder device as previously described [22]. A carotid pressure cuff is applied over the neck to detect the right carotid artery and a pressure cuff is placed around the right upper thigh to detect the femoral artery. The distance from the suprasternal notch to the middle of the thigh cuff was measured. The signal from each cuff is analysed to derive arterial transit time. PWV is calculated using the software, dividing arterial transit time by measured distance.

### Statistical analysis

GraphPad Prism (San Diego, CA, USA) was used to plot the data as mean percentage relaxation, with error bars representing standard error of the mean (SEM), and n being the number of arteries from different patients. The software was used to fit sigmoidal concentration-response curve to the mean data using a logistic equation. Percentage maximal vasorelaxant responses (Rmax) and potency (EC_50_) were calculated from this mean data were compared control and HD patients using unpaired Students' t-test, with p<0.05 taken as significant.

## Results

There was no difference in the characteristics of HD and non-HD patients other than uremic status (see Table 1). Haemodialysis is generally commenced at an eGFR below 10 mL/min/1.73 m^2^, so patients will have a lower eGFR than the controls by virtue of being on dialysis.

**Table 1:** Physical characteristic of HD patients and controls (non HD subjects).

Characteristic	HD (n = 11)	Control (n = 26)	P value
**Age (years)**	62±16	64±11	0.936
**Sex**	M = 8, F = 3	M = 24, F = 2	
**Systolic BP (mmHg)**	142±11	134±17	0.781
**Diastolic BP (mmHg)**	78±11	81±10	0.892
**MAP**	110±8	99±7	0.356
**Creatinine (µmol/L)**	NA	85±6	
**eGFR (ml/min/1.73 m^2^)**	NA	84±5	
**BMI**	28±2.8	26±2.3	0.746
**Smoker**	n = 3	n = 10	
**IHD**	n = 2	n = 1	
**CVD**	n = 0	n = 1	

Values are Mean ± SEM. *P* value indicates the comparison between the groups by t-test, and the significance at P<0.05. **Abbreviations**: eGFR, estimated glomerular filtration rate; BP, blood pressure; MAP, main arterial pressure; BMI, body mass index; IHD, ischemic heart disease; CVA, cerebrovascular accident; NA, not available; NS, non-significant.

### Effects of HD on contractile responses

In vasoconstrictor studies, the average internal diameter (ID) of arteries used was similar between HD and control groups for smaller (HD; 473±42 µm (n = 30 individual segments) *cf* control: 449±79 µm (n = 45)) or larger arteries (HD; 738±96 µm (n = 27) *cf* control: 717±89 µm (n = 40)).

In arteries less than 600 µm in diameter, the maximum KPSS contraction was greater in the HD group; 9.8±1.2 mN in HD (n = 11) and 6.99±0.32 mN in controls (n = 26), P<0.05, Students t test with Welch's correction. There was no significant difference in the mean KPSS response in arteries with an ID greater than 600 µm; 14.7±1.2 mN in HD (n = 11) and 13.0±1.2 mN in controls (n = 26).

**Figure 1:**
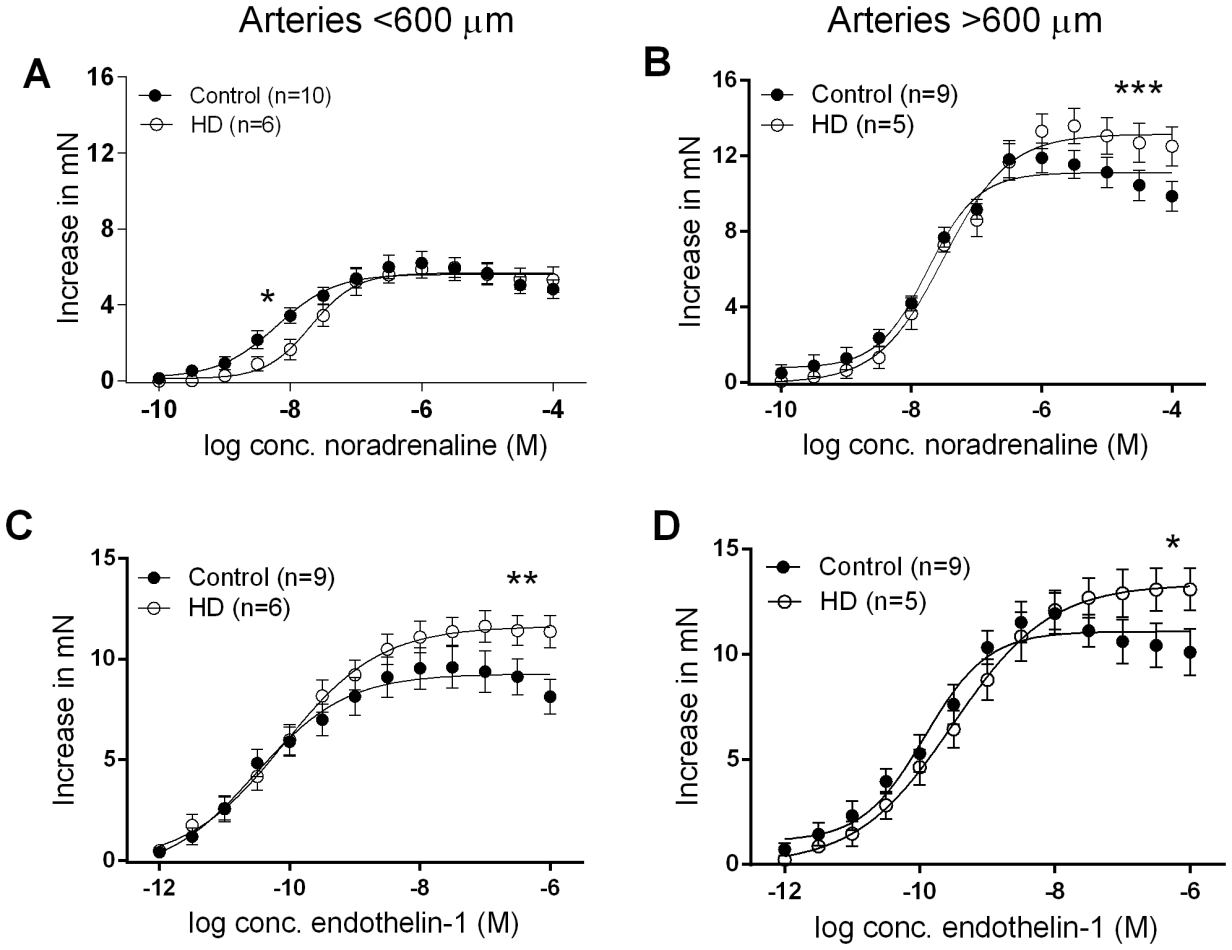
Concentration-response curves to NA (A,B) and ET-1 (C,D) in isolated subcutaneous arteries obtained from HD patients and controls. Data are expressed as mean ± SEM and the comparison is by students t-test; *P<0.05, **P<0.01, ***P<0.001. HD, haemodialysis; n, number of arteries; M, molar; mN, milliNewtons.

**Figure 2:**
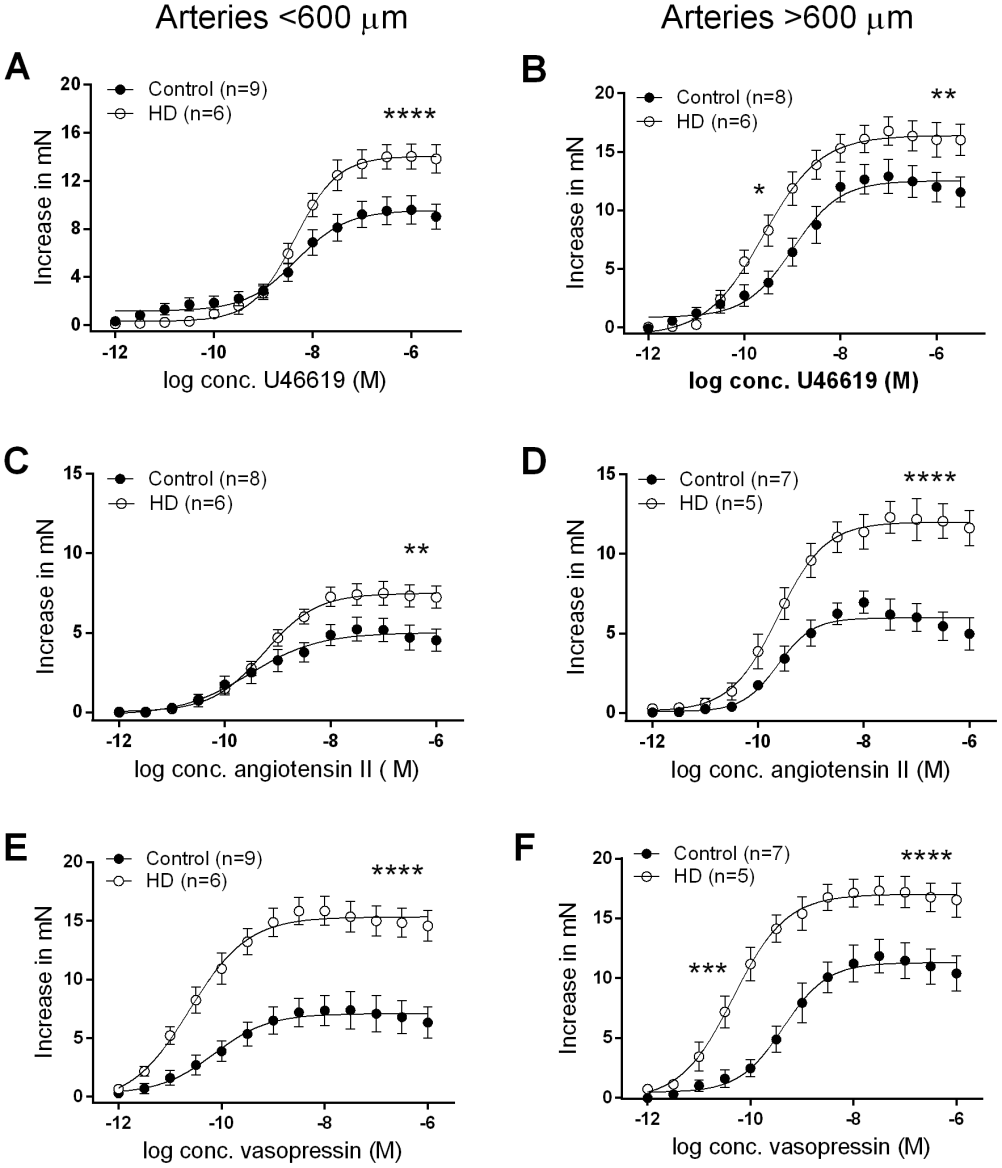
Concentration-response curves to U46619 (A,B), Ang II (C,D), and vasopressin (E,F) in arteries obtained from HD patients and controls. Data are expressed as mean ± SEM and the comparison is by students t-test; **P<0.001, ***P<0.001, ****P<0.0001. HD, haemodialysis; U4, U46619; n, number of arteries; M, molar; mN, milliNewtons.

In arteries less than 600 µm in diameter, the potency (EC_50_) of vasoconstrictor agents were similar between groups, with the exception that the potency of NA was reduced in HD patients (P<0.05, Fig. 1A). However, the maximal contractile response to ET-1 (*P*<0.01, Fig. 1A), U46619 (P<0.0001, Fig. 2A), Ang II (P<0.01, Fig. 2C) and vasopressin (P<0.0001, Fig. 2E) were all significantly enhanced in arteries from HD patients.

In arteries greater than 600 µm in diameter, the enhanced vasocontractility in HD patients was even more pronounced. The R_max_ to NA (P<0.001, Fig. 1B), ET-1 (P<0.05, Fig. 1D), U46619 (P<0.01, Fig. 2B), Ang II (P<0.0001, Fig. 2D) and vasopressin (P<0.0001, Fig. 2F) were all enhanced in HD patients. In these larger arteries, the potency of U46619 (P<0.05, Fig. 2B) and vasopressin (P<0.001, Fig. 2F) was also enhanced in subcutaneous arteries obtained from HD patients.

### Effects of HD on endothelium-dependent vasorelaxant responses

In a separate set of studies, vasodilator responses were measured in fresh arterial segments. The average ID of arteries less than 600 µm was 478±81 µm in HD (n = 18) and 512±44 µm in controls (n = 27). In arteries greater than 600 µm, it was 761±62 µm (n = 15) and 744±91 µm (n = 25) in HD and controls respectively.

**Figure 3:**
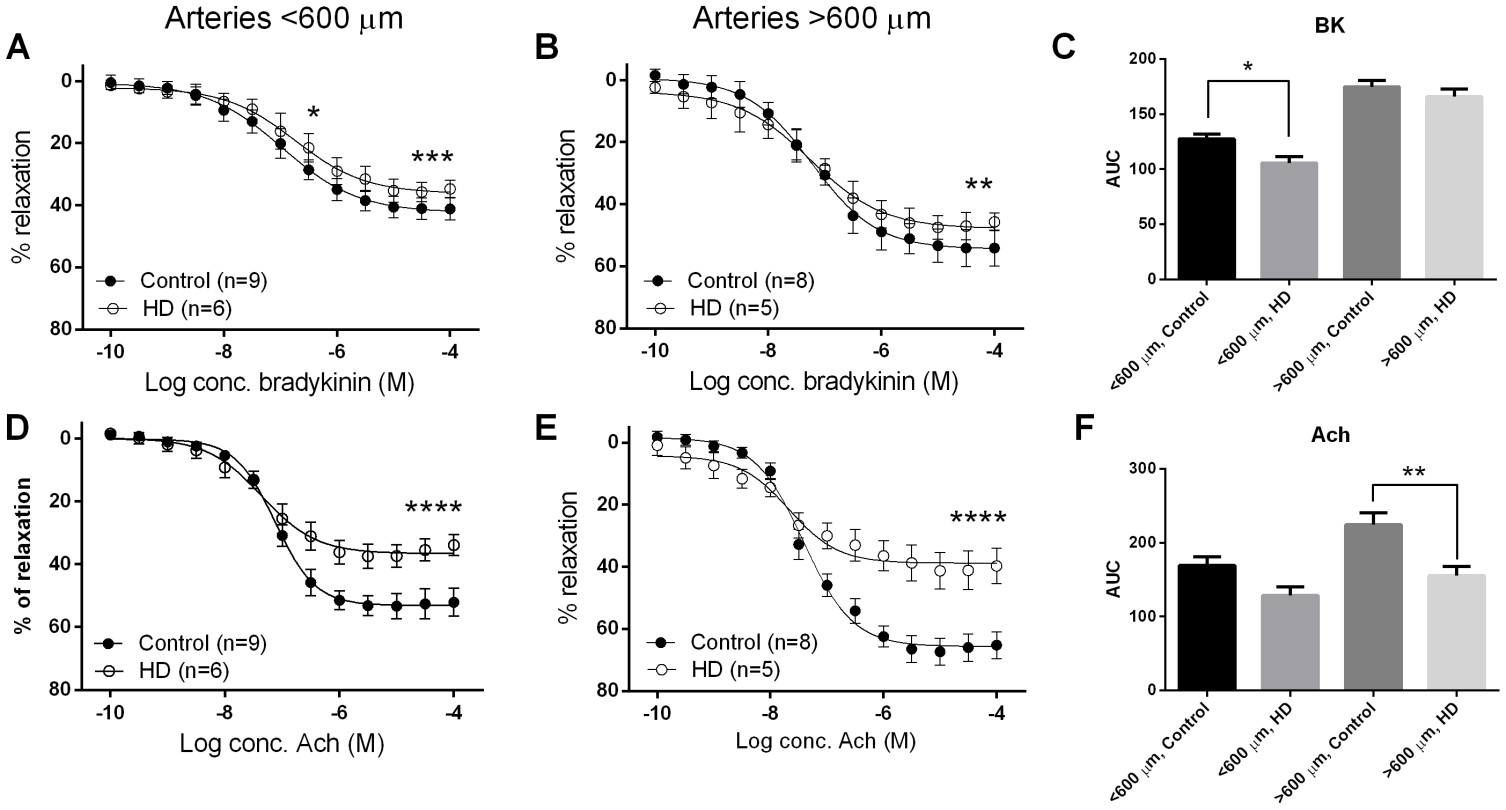
Concentration-response curves to bradykinin (A,B,C) and acetylcholine (D,E,F) in arteries obtained from HD patients and controls. Data are expressed as mean ± SEM and the comparison is by students t-test; *P<0.05, **P<0.01, ***P<0.001, ****P<0.0001. HD, haemodialysis; n, number of arteries; M, molar.

In arteries less than 600 µm, the potency of BK was reduced (P<0.05) in arteries from HD patients. The maximal vasorelaxant response to BK (P<0.001) and ACh (P<0.0001) was also significantly lower in the HD group compared to controls (Figs. 3A,B). Similarly, in arteries greater than 600 µm, significantly lower vasorelaxation to BK and ACh were obtained in HD patients compared to controls (Figs. 3D,E). The effect of HD was most pronounced on the ACh response in arteries greater than 600 µm, as evidenced by the greatest reduction in the overall response (area under the curve, AUC) (see Fig. 3C,F).

### Effects of HD on endothelium-independent vasorelaxant responses

**Figure 4:**
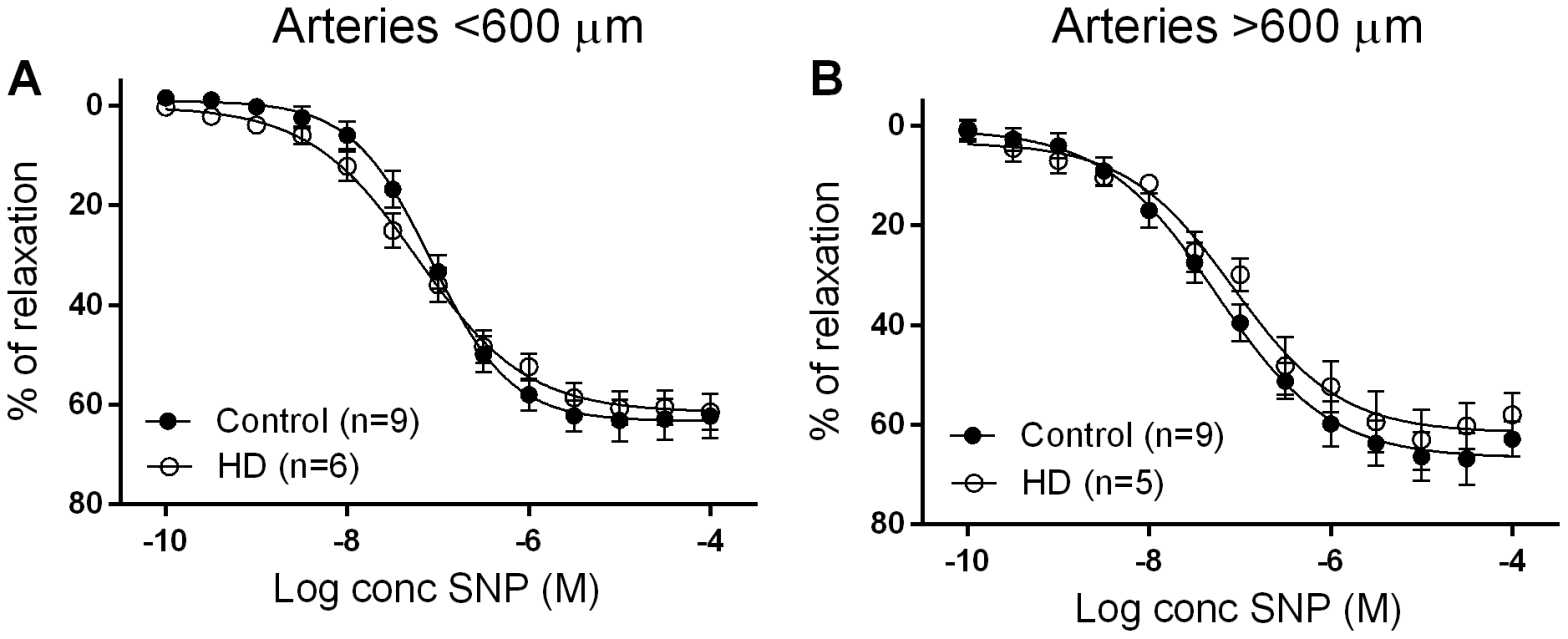
Concentration-response curves to sodium nitroprusside in arteries obtained from HD patients and controls. Data are expressed as mean ± SEM and the comparison is by student's t-test. HD, haemodialysis; n, number of arteries; M, molar.

The vasorelaxant response to SNP was similar between groups in subcutaneous arteries in arteries of either sized diameter (Fig. 4).

### Correlation of *ex vivo* data with *in vivo* measurements in HD patients

PWV was significantly correlated with the maximum contractile response of arteries greater than 600 µm to vasopressin (r = 0.829, P = 0.042) (Table 2). When contractile responses were expressed as a percentage of maximum KPSS response, a positive correlation was also seen between PWV and percentage of maximum response of arteries less than 600 µm to vasopressin (r = 0.886, *P* = 0.019), see table 3. There was a trend for contractile responses to U46619 (*P* = 0.058) and Ang II (*P* = 0.083) (Table 3) in arteries greater than 600 µm to also be correlated with PWV. A negative correlation between DBP and the normalised vasopressin responses in arteries greater than 600 µm (r = −0.829, *P* = 0.042, Table 3). A negative correlation was observed between the DBP of HD patients and the response of large arteries to SNP (r = −0.974, *P* = 0.016) (Table 2).

**Table 2:** Correlations of *in vivo* cardiovascular data with *ex vivo* vasoactive responses of arteries obtained from HD patients to different stimuli.

Vasoactive agent	Vessel size	PWV		SBP		DBP	
		*r* value	*P* value	*r* value	*P* value	*r* value	*P* value
**Noradrenaline**	Small	0.377	0.461	0.492	0.355	−0.422	0.372
	Large	0.200	0.783	0.000	1.050	0.185	0.344
**Endothelin-1**	Small	0.143	0.802	−0.200	0.713	−0.388	0.252
	Large	−0.300	0.624	0.000	1.050	0.500	0.673
**U46619**	Small	0.00	1.00	−0.463	0.355	0.222	0.557
	Large	0.232	0.658	0.028	1.000	−0.177	0.344
**Angiotensin II**	Small	0.428	0.419	0.028	1.000	−0.255	0.522
	Large	0.100	0.950	−0.800	0.133	0.551	0.585
**Vasopressin**	Small	−0.486	0.329	0.542	0.297	−0.430	0.320
	Large	0.829	0.042 ^*^	−0.600	0.241	0.570	0.455
**Bradykinin**	Small	0.232	0.658	0.057	0.919	0.755	0.355
	Large	0.00	1.00	−0.800	0.133	−0.433	0.322
**Acetylcholine**	Small	−0.771	0.102	0.142	0.802	0.100	0.873
	Large	0.100	0.950	0.000	1.050	−0.233	0.644
**Sodium nitroprusside**	Small	0.543	0.297	−0.257	0.658	0.482	0.551
	Large	−0.400	0.516	0.1000	0.950	−0.954	**0.012** ^**^

**Abbreviations**: PWV, pulse wave velocity; SBP, systolic blood pressure; DBP, diastolic blood pressure; r value, correlation coefficient; *P<0.05, **P<0.01.

**Table 3:** Correlations of *in vivo* data with *ex vivo* normalised vasoconstrictor responses of arteries obtained from HD patients.

Vasoactive agent	Vessel size	PWV		SBP		DBP	
		*r* value	*P* value	*r* value	*P* value	*r* value	*P* value
**Noradrenaline**	Small	0.314	0.563	0.200	0.713	−0.200	0.713
	Large	0.700	0.233	0.200	0.783	0.200	0.783
**Endothelin-1**	Small	0.371	0.497	0.371	0.497	0.542	0.297
	Large	0.300	0.683	−0.100	0.950	−0.820	0.133
**U46619**	Small	0.428	0.419	0.600	0.241	0.428	0.419
	Large	0.828	**0.058**	0.142	0.802	−0.028	1.000
**Angiotensin II**	Small	0.314	0.563	0.085	0.919	−0.428	0.419
	Large	0.900	**0.083**	−0.400	0.516	−0.410	0.516
**Vasopressin**	Small	0.942	0.018^*^	−0.542	0.297	−0.885	0.033*
	Large	−0.371	0.497	0.200	0.713	0.600	0.208

Contractile responses to each spasmogen were normalised to the maximal contractile response produced in that arteries in response to a high potassium solution. **Abbreviation**: PWV, pulse wave velocity; SBP, systolic blood pressure; DBP, diastolic blood pressure; r value, correlation coefficient; *P<0.05.

## Discussion

Endothelial dysfunction is a crucial element in the pathophysiology of increased cardiovascular risk amongst CKD patients, including those on HD. Limited human studies reported to date [15]–[17] have examined the effect of uraemia on isolated vascular function. These studies were conducted on small arteries isolated from ESRD patients (with a very wide range of dialysis vintages), investigating a limited suit of vasoactive agents. The current study was therefore conducted on subcutaneous arteries isolated from HD patients investigating different vasoconstrictors and vasodilators. The present study provides a number of key findings. Firstly there is a markedly increased responsiveness of isolated arteries from HD patients to a range of vasoconstrictors. Secondly, impaired endothelium-dependent vasodilatation, but preserved endothelium-independent vasodilatation, is observed in arteries of HD patients. Thirdly, *ex vivo* responses of small isolated arteries were correlated to clinically relevant *in vivo* assessment of cardiovascular system function.

The data published to date on enhanced vasoconstrictor responses of isolated arteries have been somewhat contradictory. Increased contractile responses of isolated arteries have been reported before in CKD patients (not HD), characterised by a greater response to NA and ET-1 [15]. However, in a later study, similar contractile response of small arteries to NA, ET-1, and Ang II was observed between uremic and control groups [16]. This is in contrast to our study with consistently observed significant increments in the arterial contraction in isolated different-sized arteries of HD patients in response to NA, ET-1, U46619, Ang II, and vasopressin in arteries of 2 calibres. The effects of HD were most pronounced on the contractile response to U46619, angiotensin II and vasopressin, and the effect of HD was greater in the arteries of diameter greater than 600 µm, where an increased sensitivity (pEC_50_) to U46619 and vasopressin was also observed. The data derived from our study are consistent with a previous report on isolated subcutaneous arteries where increased contractions to NA and ET-1 were observed in patients with ESRD [15], but is in contrast with Aalkjaer *et al*, in which a similar response to NA and Ang II was observed between controls and patients with chronic intermittent dialysis [23]. Our population was unselected (no survivor or transplant bias) and all had been exposed to a very similar and short period of HD (<90 days), furthermore a larger number of arterial segments were studied with the benefits of robust normal volunteer comparators.

Interestingly, our data showed differential effects of HD on contractile responses to the various agents with some ligands (U46619, angiotensin II and vasopressin) being more affected than others (noradrenalin and endothelin-1). This suggests that there is not a global upregulation of contractile function intrinsic to smooth muscle, and must relate to either the expression or function of the receptors involved, or the intracellular mechanisms to which the receptors are coupled. Since all the receptors activated by these ligands are Gq- coupled (bringing about contraction via increased IP_3_ and DAG levels is to increase intracellular Ca^2+^), changes in the expression of the receptors seems a more likely explanation.

In addition to excessive pressor response of isolated arteries from HD patients, all showed significant decreased endothelium-dependent vasodilatation. This defect was observed in both artery sizes in HD patients, although the blunted response to acetylcholine was bigger in the arteries with a larger diameter. By contrast, the vasorelaxant response to SNP (endothelium-independent vasodilator) was preserved in the HD group. Previous studies on isolated subcutaneous arteries of uremic ESRD patients have similarly demonstrated impaired vasodilatory function in response to ACh but not SNP [15], [16]. Luksha *et al.* also found that the percentage relaxation and the sensitivity (EC_50_) to both BK and ACh were reduced in isolated small arteries of patients commencing PD [17]. Similar results have been reported previously *in vivo* using non-invasive techniques such as Doppler ultrasound [13], and forearm plethysmography [11]. Together this suggests that endothelial-dependent dysfunction is widespread in CKD, observed in conduit arteries, as well as the microcirculation.

This impaired relaxation response could be due to NO dysfunction or decreased ability of vascular endothelium to produce and release vasodilator substances such as endothelium-derived hyperpolarizing factor (EDHF). These vasodilators act by stimulating release of both NO and NO-independent factor (possibly EDHF), from human vascular endothelium [24]. Circulating uremic factors in HD patients may responsible for these changes. These toxins have being widely considered and include elevated plasma homocysteine level [25], increase reactive oxygen species [7], [8], and elevated plasma levels of nitric oxide synthase inhibitors such as ADMA in dialysis patients [10], [26]. In our study, arteries were examined after being removed from exposure to humeral consequences of CKD, suggesting adaptive responses have occurred within the arteries, and the blunted responses are not just a consequence of circulating factors.

Interestingly, the effects of HD were more pronounced when looking at the relaxant response to ACh than BK, and in the larger than smaller calibre arteries. Endothelium-dependent relaxations are achieved by a combination of endothelium-derived prostaglandins, NO, and EDHF. At least for ACh, the role for NO is thought to be more important in conduit arteries and the role for EDHF more important in distal resistance vessels. Thus the greater blunting of ACh responses in arteries greater than 600 µm in our study could reflect a greater contribution of NO production in these arteries, and point to defective NO production as a mechanism of action for the blunted endothelium-dependent responses in the HD patients.

This study represents the first attempt to examine any association between *in vivo* cardiovascular performance and *ex vivo* arterial function. PWV positively correlated with a greater response to vasopressin, and there was a trend for an association with the enhanced responses to U46619 and angiotensin II also. It is of note, that these are also the ligands whose responses were enhanced more by HD, and suggests the enhanced responses to these ligands play contribute to the increased PWV seen in HD patients. In addition, the present study found that the DBP was inversely correlated with the contractile response of small arteries to vasopressin, and negatively correlated with the vasorelaxation response of large arteries to SNP. The existence of overhydration in those dialysis patients which have been identified as hypertensive is far from universal. Wabel and co-workers measured pre-dialysis SBP and fluid status in 500 HD patients by bioimpedance monitoring (compared to a matched healthy population). Only 15% of patients fitted the stereotype of fluid overload with hypertension. 13% of patients had hypertension despite dehydration and 10% had fluid overload despite normal BP or hypotension [27]. Similar findings were seen in a another study using the same methodology in 639 patients using PD [28]. These studies highlight that although physicians often estimate hydration status by BP, the two factors are often dissociated in the setting of the profound physiological derangement characteristic of dialysis dependant CKD. A wide variety of potential other pathophysiological processes characteristic of dialysis patients may contribute to generating hypertension without recourse to simple fluid overload, and enhanced pressor response of abnormal uremic resistance vessels appears important.

In conclusion, this study provides additional insights into the effect of HD on vascular function. Exaggerated pressor response and endothelium based failure of relaxation are associated with *in vivo* measurements of markers of cardiovascular performance we already know to be important in determining HD patient survival. This defective vascular response may be important in sensitising HD patients to recurrent cumulative ischaemic end organ injury driven by the circulatory stress of HD.
